# The effect of the optical design of multifocal contact lenses on choroidal thickness

**DOI:** 10.1371/journal.pone.0207637

**Published:** 2018-11-16

**Authors:** Katharina Breher, Miguel García García, Arne Ohlendorf, Siegfried Wahl

**Affiliations:** 1 Institute for Ophthalmic Research, Eberhard Karls University Tuebingen, Tuebingen, Germany; 2 Carl Zeiss Vision International GmbH, Aalen, Germany; Massachusetts Eye & Ear Infirmary, Harvard Medical School, UNITED STATES

## Abstract

Studies have found reduced myopia progression with multifocal contact lenses, albeit with an unclear mechanism behind their protective effect. It is hypothesized that the induced myopic defocus of the addition zones of the multifocal contact lenses leads to choroidal thickening and therefore inhibits eye growth. In the current study, the effect of the optical design of multifocal contact lenses on choroidal thickness was investigated. Eighteen myopic participants wore four different contact lenses ((1) single-vision lenses corrected for distance, (2) single-vision lenses with +2.50 D full-field defocus, (3) Multifocal center-distance design, (4) Multifocal center-near design, both with addition power +2.50 D) for 30 min each on their right eye. Automated analysis of the macular choroidal thickness and vitreous chamber depth were performed before and after the wear of each of the contact lenses. Peripheral refraction profiles in primary gaze were obtained using eccentric photorefraction prior to contact lens wear. Choroidal thickness and vitreous chamber depth showed no significant differences to baseline with any of the contact lenses (all p > 0.05). Choroidal thickness increased by +2.1 ± 11.1 *μ*m with the Multifocal center-distance design, by +2.0 ± 11.1 *μ*m with the full-field defocus lens, followed by the Multifocal center-near design with +1.6 ± 11.3 *μ*m and the single-vision contact lens correcting for distance with +0.9 ± 11.2 *μ*m. Multifocal contact lenses have no significant influence on choroidal thickness after short-term wear. Therefore, changes in choroidal thickness might not be the main contributor to the protective effect of multifocal contact lenses in myopia control.

## Introduction

Currently, the worldwide prevalence of myopia constantly continues to increase, with predictions of half of the population being myopic by the year 2050 [1]. Myopia results in distance blur despite inactive accommodation as the light focuses in front of the retina, mainly due to an elongated ocular axial length [2, 3]. Due to the absence of scleral innervation, the choroid assumingly plays a major mediating role in the regulation of ocular growth, possibly as a reaction to defocus. Hereby, the signal cascade from the retinal neurons must pass through the choroid on the way to the sclera [4]. On the other hand, the choroid is capable of directly influencing the scleral biochemical properties by secreting various growth factors and matrix metalloproteinases [5].

To delay the onset and slow the progression of myopia, research has been evaluating various optical and pharmacological control interventions [6]. The optical strategies include spectacle lens designs, such as progressive addition, bifocal and radial-refractive gradient lenses, as well as contact lens solutions, such as bifocal, multifocal and orthokeratology contact lenses. Multifocal contact lenses (Proclear Multifocal “D”; CooperVision, Inc., Fairport, NY, USA) have been shown to efficiently prevent myopia progression up to 50% in children regarding their refractive error development during two years of wear [7]. These interventions altogether aim to reduce hyperopic defocus, especially on the peripheral retina [8] As shown in animal studies, full-field and locally imposed hyperopic defocus results in compensatory axial eye growth and thus faster myopia progression [9–11].

In experiments with chicks, for example, the imposition of negative lenses creating hyperopic retinal defocus leads not only to axial elongation but also choroidal thinning, and vice versa, for myopic defocus with associated choroidal thickening [11]. Studies on humans with unilateral full-field spectacle lens defocus found similar axial and choroidal reactions [12–14]. The imposition of myopic full-field defocus with contact lenses instead of spectacle lenses also leads to choroidal thickening. The amplitudes of thickness changes range from 2 *μ*m up to 15 *μ*m for different defocus power and time conditions [15, 16]. Furthermore, the effect seems to be more pronounced with myopic defocus than hyperopic defocus, and more pronounced on the nasal choroid [15, 16].

While choroidal thickness changes after exposure to full-field defocus have been shown in the aforementioned human studies, there are no results about choroidal reactions to multifocal contact lenses with different refractive profiles. To the best of our knowledge, this is the first study assessing the impact of two oppositely designed multifocal contact lenses on the human choroidal thickness and vitreous chamber depth.

## Materials and methods

### Subjects

This prospective main study, as well as the complementary prestudy, was carried out at the University of Tuebingen. The study protocols followed the Declaration of Helsinki 1964 and following amendments, as well as the data protection regulations. The study was approved by the ethics committee of the Faculty of Medicine of the University Tuebingen. Written informed consent was obtained from all subjects prior to the measurements. Participants between 18 and 35 years of age were included, with a spherical equivalent refraction (SE) between 1.00 D and 6.00 D, maximal cylindrical power of (+ or -) 1.00 D and visual acuity of 0.1 logMAR or better, when corrected with the SE only. Furthermore, Bruch’s membrane and the choroidal-scleral interface in the optical coherence tomography (OCT) scan had to be sufficiently detectable to ensure a correct analysis of the choroidal thickness (ChT) of the OCT scan by the automated choroid segmentation. Hereby, the intrasubject repeatability of the measurements had to be 15 *μ*m or better as an additional inclusion criterion, since this was the limit for qualitative good choroid segmentation, as observed in the small subset of the first complementary study.

### Contact lenses

Table 1 lists the types and parameters of the applied contact lenses. The defocus power and multifocal addition were each +2.50 D based on the subject’s individual refraction. The Proclear Multifocal ‘D’ (MFD) lens has a center-distance design with a 2.3 mm diameter zone of distance correction. In the progressive zone, up to 8.5 mm diameter, the power slowly increases until it reaches the full addition power. The outermost ring has no optical effect because it lies outside the pupil [7, 17]. In contrast, the Proclear Multifocal ‘N’ (MFN) lens is structured oppositely with a central ring that consists of the maximum addition power and a progressive power decrease to the distance correction within the transition zone [17]. Both monofocal contact lenses, one with distance correction (SVDC) and one with a full-field undercorrection (SVDF), were used as a comparison to the multifocal contact lenses.

**Table 1:** Applied contact lenses with parameters.

Parameter	Single vision distance lens (SVDC)	Single vision defocus lens (SVDF)	Multifocal center distance lens (MFD)	Multifocal center near lens (MFN)
Commercial name	Proclear	Proclear	Proclear multifocal ‘D’ Add +2.50 D	Proclear multifocal ‘N’ Add +2.50 D
Base curve (mm)	8.6	8.6	8.7	8.7
Diameter (mm)	14.2	14.2	14.4	14.4
Material	omafilcon B	omafilcon B	omafilcon B	omafilcon B
Water content (%)	62	62	62	62
O_2_ permeability (Dk/t at -3.00 D)	42	42	17	17

Abbreviations of the contact lenses in brackets in the first row are used hereinafter in the text.

### Main study protocol

**Fig 1:**
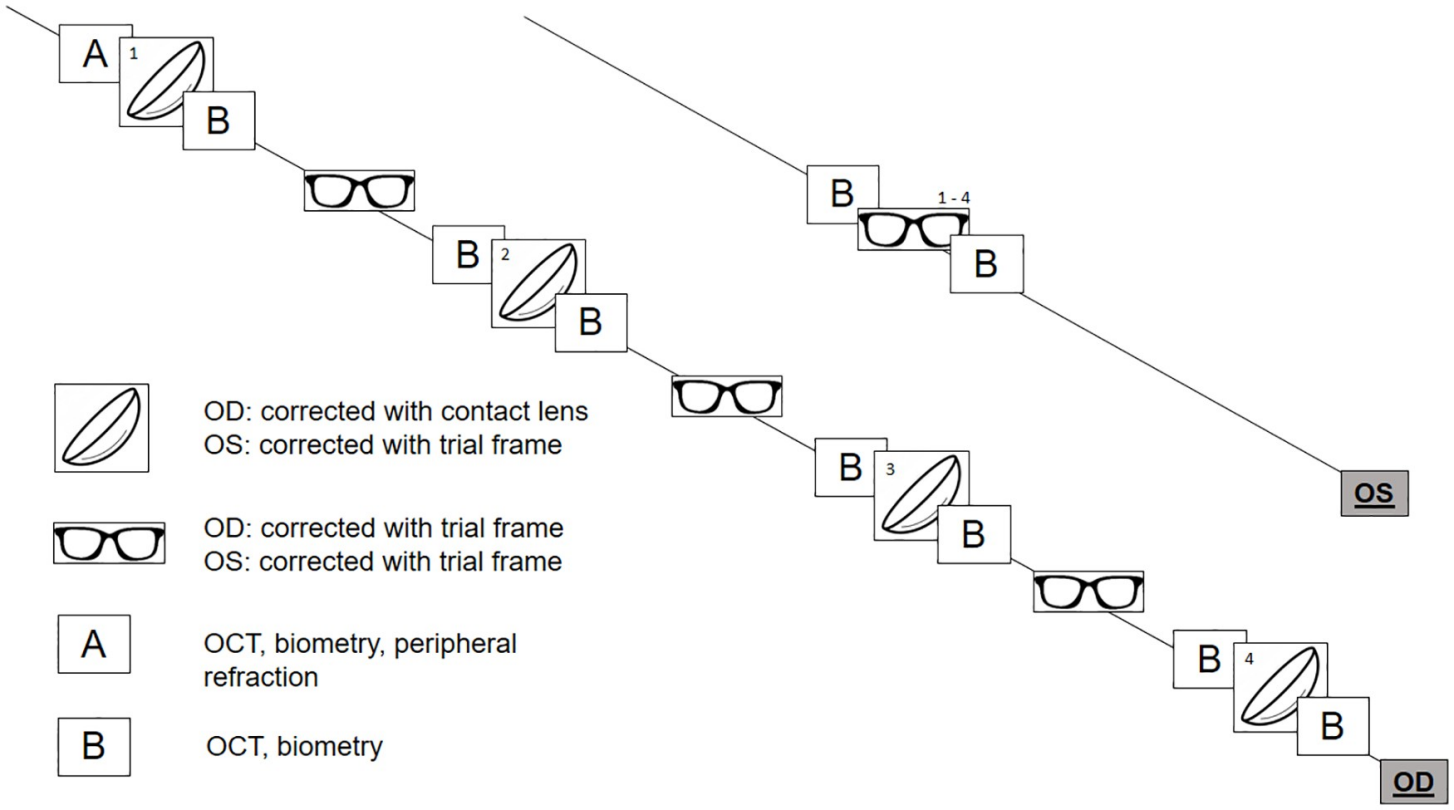
Study procedure of the main study. Subjects underwent OCT and biometry measurements before and after 30 min of contact lens wear. This was followed by a 15 min wash-out period with trial frame correction. The procedure was repeated for each of the four contact lenses. The left eye was constantly wearing trial frame correction while undergoing the same measurements at the same time points as the right eye.

The protocol of the main study consisted of OCT scans (CIRRUS HD-OCT 5000, Carl Zeiss Meditec Inc., Dublin, CA, USA) and biometry measurements (IOLMaster 700, Carl Zeiss Meditec AG, Jena, Germany) before and after 30 min of wearing each of the four different contact lenses. In addition, peripheral refraction profiles in primary gaze were obtained using photorefraction. The refractor used a moving mirror to measure refractive errors of both vertical and horizontal meridians out to an eccentricity of ±50° nasally and temporally as described previously [18]. The fixation target for peripheral refraction was mounted at a distance of 3.50 m. The lenses were worn in the right eye, while the left eye was fully corrected for distance with a trial frame and served as the control eye. After each contact lens was worn for 30 min, OCT and biometry scans were performed three times on both eyes, whereas peripheral refraction was performed six times on the right eye only. A 15 min wash-out period with trial frame correction on both eyes was executed between the single contact lenses. The study procedure is shown in Fig 1. During each round of contact lens wear, subjects watched a movie at a distance of 4.50 m (diagonal screen size 55”) in a room illuminated with 25 lux, to ensure a pupil size of at least 5 mm. The order of contact lenses, as well as the order of the devices used for the described measurements, were randomized. The anterior segment picture of the IOLMaster 700 was used for the control of the contact lens centration on the eye. All participants were scheduled between 1 pm and 3 pm, to diminish intersubject differences in ChT changes due to the diurnal choroidal rhythm [19, 20].

### Data analysis and statistics

Choroidal segmentation and thickness analyses were performed automatically with custom MATLAB (MATLAB 2017b, The MathWorks, Inc., Natick, MA, USA) software for choroid segmentation [21]. The obtained 6x6 mm^2^ Macular Cube OCT scan was presented as a squared 2D-matrix, which consisted of single values representing the choroidal thickness of every pixel location in the cubic scan. This map of three averaged measurements was used for the choroidal thickness analysis and was further sectioned into the nine ETDRS fields within MATLAB. The foveola, with its corresponding subfoveal thickness values, was therefore set into the center of the 6x6 mm^2^ OCT scan, as well as in the corresponding segmentation matrix. The OCT scan area covered a retinal angle of 20° for an axial length of 24 mm. In addition to the built-in eye tracking software, the pixel values around the foveola were averaged with the size of a regular microsaccade [22] for the calculation of subfoveal choroidal thickness. To obtain the difference in choroidal thickness from the corresponding baseline measurement (three averaged measurements each), the values before contact lens wear were subtracted from the values after contact lens wear. Positive numeric values indicate an increase in ChT, whereas negative values indicate a decrease. For each subject, the differences in choroidal thickness of one ETDRS region in the right eye were compared to the corresponding values of the same region in the left eye (control eye). Statistical analysis in MATLAB consisted of the Kruskal-Wallis test, which represents the nonparametric version of the one-way ANOVA.

Vitreous chamber depth was calculated in Excel (Microsoft Excel 2013, Microsoft Corporation, Redmond, WA, USA) by subtracting the corneal thickness, anterior chamber depth and lens thickness from the axial length. The normally distributed differences between treated and control eyes were compared with one-way ANOVA.

For the analysis of peripheral refraction, the mean of the dioptric values in the horizontal and vertical meridian were taken as SE at each angle point in steps of 0.71° for ± 50° from the fovea. The corresponding values of at least six scans were averaged, to obtain the refractive profile of the uncorrected eye.

The adjustment of the critical p-value for all analyses was performed with the Benjamini & Hochberg / Yekutieli familywise error posthoc correction [23].

### Complementary study

Within a small subset of n = 3 subjects, a complementary prestudy was conducted. Materials, measurement devices and data analysis modalities were the same as described in the main protocol, except for the ETDRS grid, since only the 1 mm central field was evaluated in this study. Subjects’ choroidal thickness of both eyes was measured on three separate days in 2 h intervals between 9 am and 5 pm while wearing regular spectacle distance correction lenses during the day. The purpose was to define the time of the day when the choroid thins in its diurnal rhythm for the scheduling of the participants. On two additional days, the subset wore each of the multifocal contact lenses on both eyes to estimate the maximal effect size of choroidal thickness change with multifocal contact lenses after day-long wear compared to a shorter 30 min exposure in the main study protocol.

## Results

The main study investigated the short-term influence of multifocal contact lenses on choroidal thickness (ChT) and vitreous chamber depth. In contrast, the complementary study evaluated the choroidal diurnal rhythm and the induced long-term effects of multifocal contact lenses.

### Baseline values

The mean age of the 18 included the participants from the main study was 25 ± 3 years, with an SE refraction of -3.8 ± 1.3 D and -3.8 ± 1.4 D, for the right and left eyes respectively. The subset of the 3 subjects from the complementary study had a mean age of 27 ± 3 years and an SE refractive error of -4.8 ± 1.0 D in the right eye and -4.8 ± 0.8 D in the left eye.

The average repeatability for the choroidal thickness measurement and analysis for all subjects was 10.8 ± 3.7 *μ*m for the right eye and 9.6 ± 6.2 *μ*m for the left eye. Prior to the experiment, there was no difference between the ChT of both eyes at any retinal region divided by the Early Treatment Diabetic Retinopathy Study (ETDRS) [24] (all p > 0.05). The choroid was thickest centrally with 220.7 ± 44.5 *μ*m and superiorly with 227.4 ± 32.2 *μ*m, whereas the inferior and nasal regions were thinnest with values of 206.0 ± 43.6 *μ*m and 149.2 ± 39.8 *μ*m, respectively (data reported only from right eye).

There were no significant correlations between ChT and the myopic refractive error or ChT and vitreous chamber depth at any location. However, the vitreous chamber depth and the magnitude of myopia showed a significant negative correlation (Pearson R = -0.54, p < 0.001).

**Fig 2:**
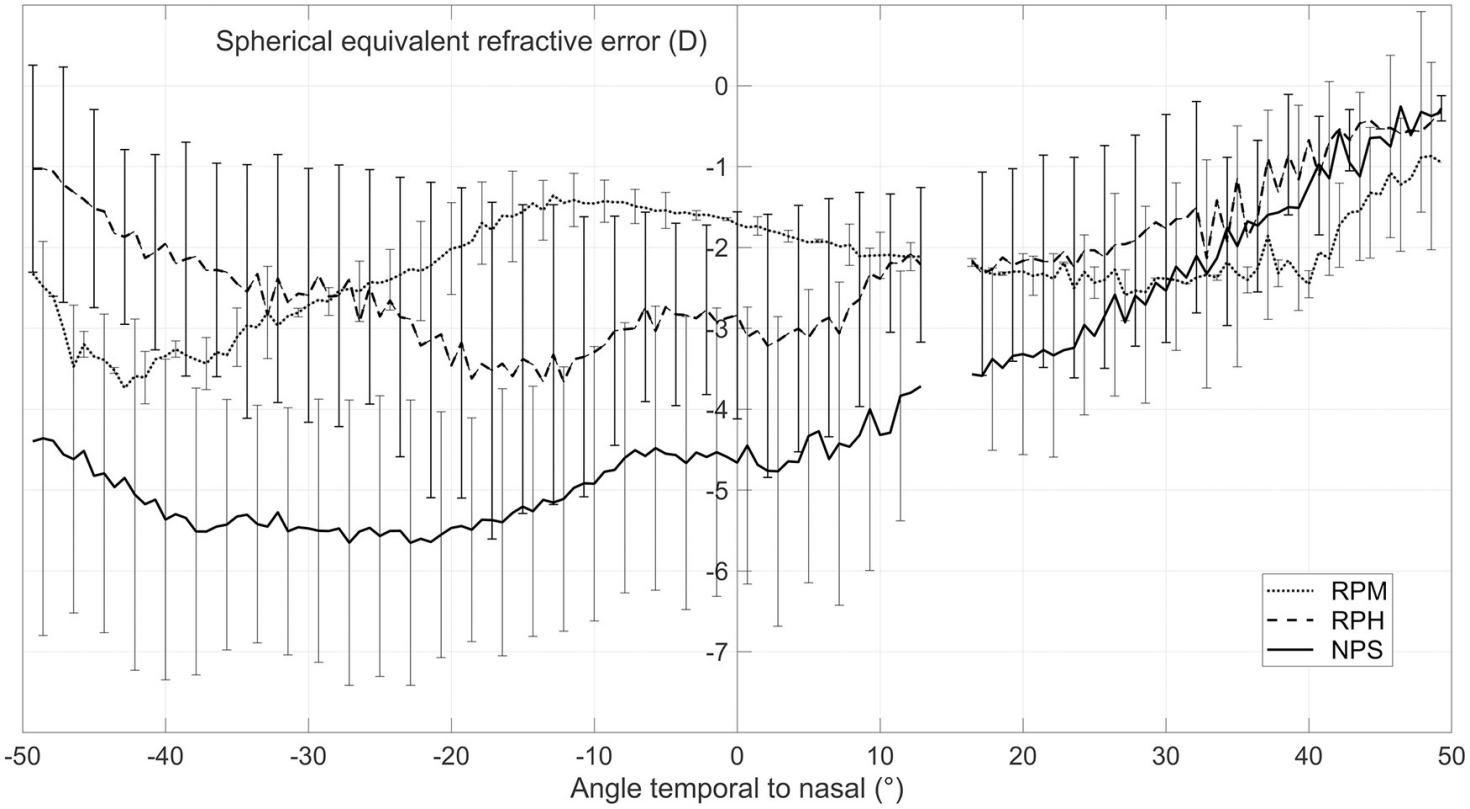
Uncorrected peripheral refraction. The average uncorrected eccentric refraction profiles are divided into three groups: NPS, RPH and RPM. Visualization clearly shows the distinct behavior of the peripheral refractive error compared to the central refractive error of the different groups. Error bars indicate the standard deviation and are plotted only for every third angle of measurement for reasons of clarity.

Eccentric photorefraction revealed three main groups of uncorrected refractive profiles. The n = 5 participants (28%) with relative peripheral hyperopia (RPH) showed relative hyperopic shifts in the periphery compared to their on-axis myopia. Relative peripheral myopia (RPM) with the opposite relationship was measured in two subjects (11%) only. The majority (n = 11 subjects, 61%) revealed a skewed profile (NPS) with relative hyperopia nasally and more myopia temporally. These groups are presented in Fig 2. The RPM group revealed the least myopic central refractive error with -1.73 ± 0.08 D, followed by the RPH group with -2.92 ± 1.32 D. The subjects with NPS profiles were measured with the highest on-axis myopia of -4.49 ± 1.68 D.

### Diurnal rhythm of choroidal thickness and maximum effect of day-long multifocal contact lens wear

**Fig 3:**
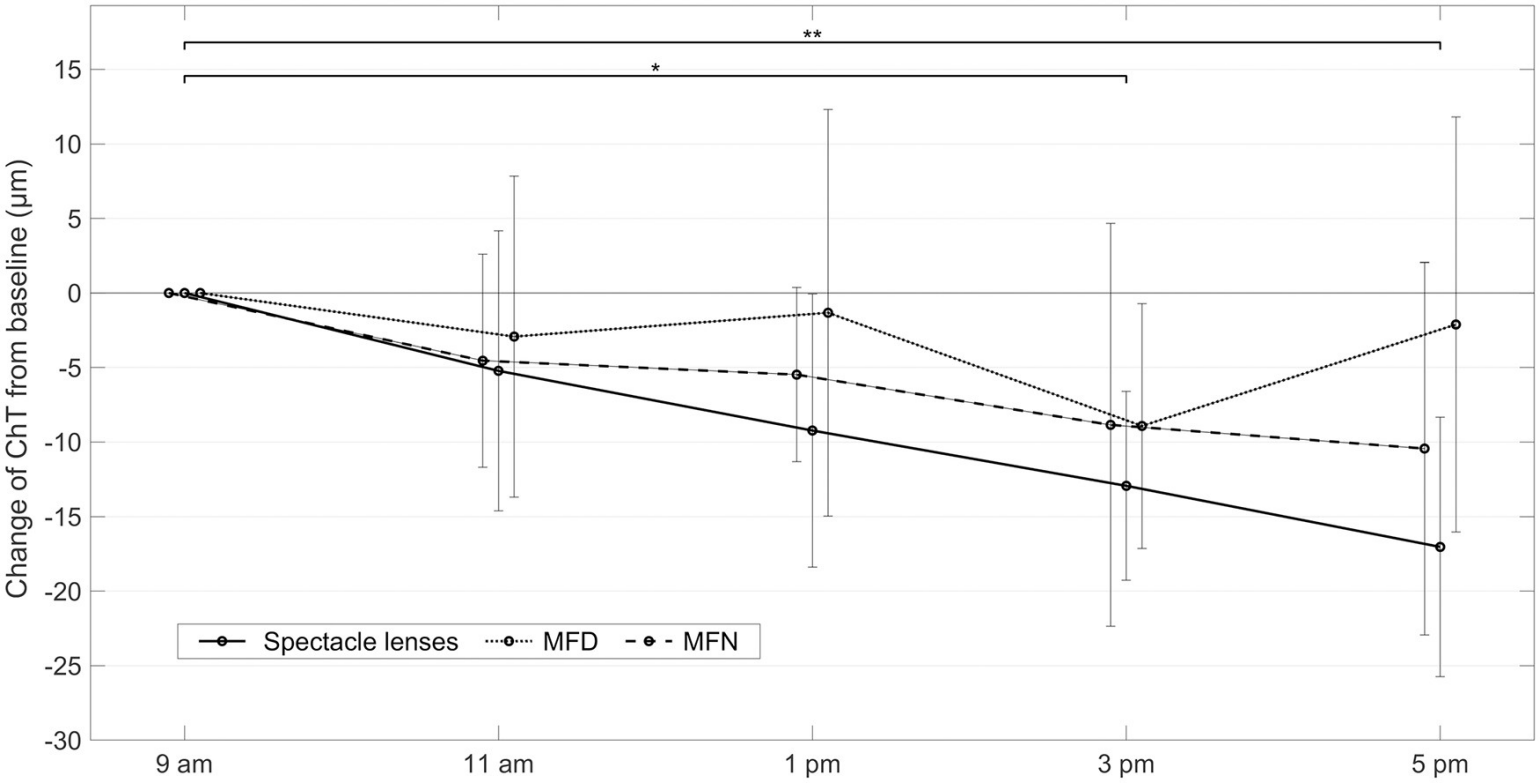
Diurnal choroidal rhythm with different corrections. Central choroidal thickness changes for n = 3 subjects from 9 am to 5 pm. The ChT in the afternoon was significantly thinner compared to the morning values only with the spectacle correction only (9 am to 3 pm: p = 0.03; 9 am to 5 pm: p = 0.008). There was less thinning with both multifocal contact lenses. The horizontal offset of the values at each time point is only for reasons of clarity. Errorbars indicate the standard deviation.

The central choroid constantly decreased in thickness over the course of the day when wearing regular spectacle distance correction lenses (Fig 3). At 3 pm and 5 pm, it was significantly thinner than in the morning with a relative thickness decrease from baseline of 12.9 ± 6.3 *μ*m at 3 pm (p = 0.03) and 17.0 ± 8.7 *μ*m at 5 pm (p = 0.008). To account for this diurnal pattern in the planning of the main study, the subjects were scheduled for the experiment at the times with thinning choroids, starting between 1 pm and 3 pm. There was also choroidal thinning present with both multifocal contact lenses but less pronounced and constant compared to the course with spectacle lens correction. None of the diurnal ChT changes while wearing multifocal contact lenses reached a significant difference from the morning values (all p > 0.05).

### Choroidal thickness changes after short-term wear of multifocal contact lenses

**Fig 4:**
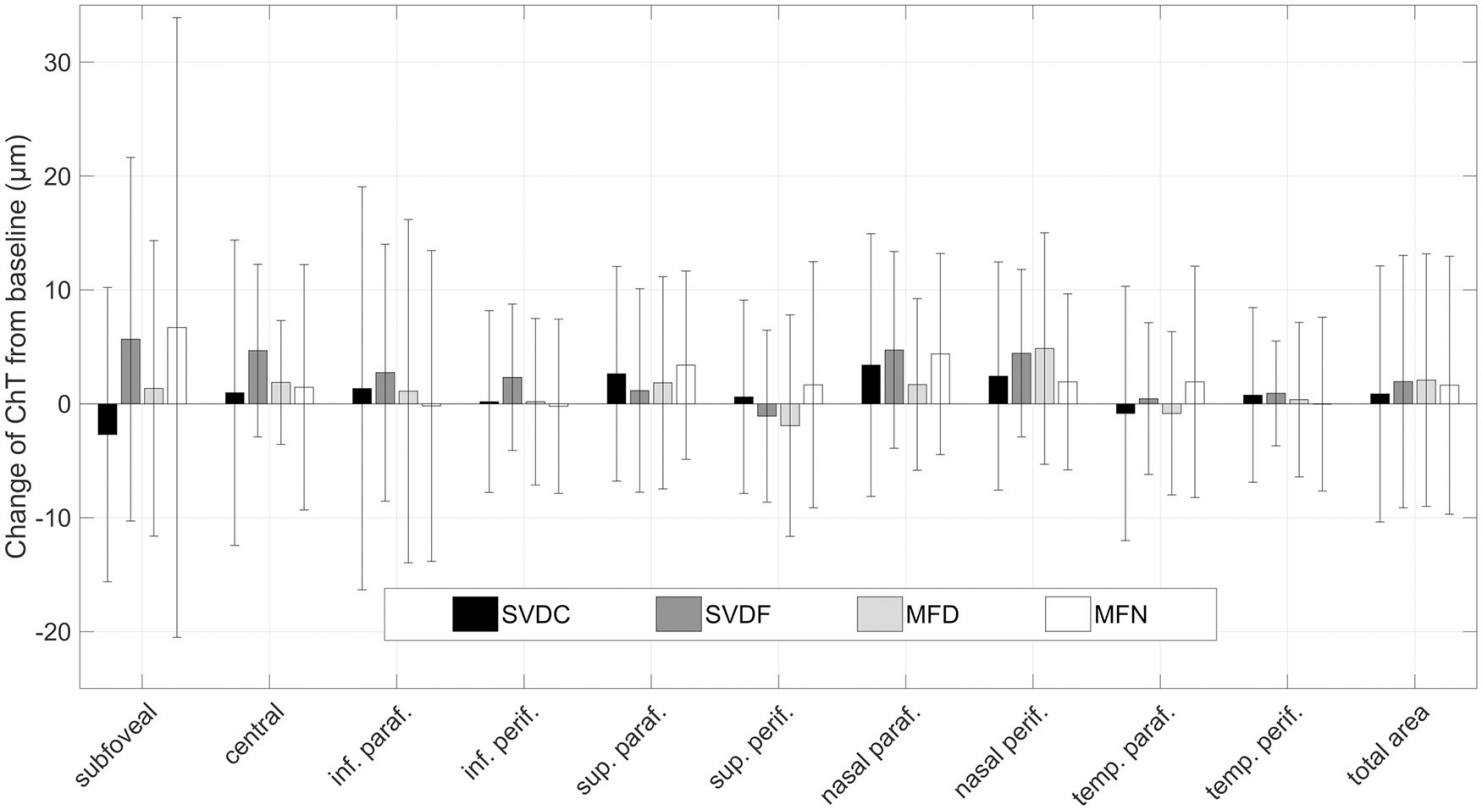
Absolute choroidal thickness changes with different lenses. Absolute ChT changes of the right eye with different contact lenses, split by ETDRS regions and averaged over the total macular area. The error bars indicating the standard deviation imply a highly individual variability in choroidal reactions.

The difference of the measurement values in choroidal thickness and vitreous chamber depth before and after each 30 min period with a certain lens were considered as lens-induced changes. Although the absolute changes in choroidal thickness of the right eye for every ETDRS region and the applied contact lenses were not statistically significant (all p > 0.05), subtle trends were visible and are displayed in Fig 4. In the subfoveal region, the SVDC lens generally led to choroidal thinning (-2.7 ± 12.9 *μ*m) compared to the more pronounced thickening with the SVDF and MFN lenses (+5.7 ± 16.0 *μ*m and +6.7 ± 27.2 *μ*m). However, the amplitude of the reaction matched their power structures, since both of their centers were defocused with +2.50 D. When averaged over the total scan area (see last column in Fig 4), most choroidal thickening resulted from the full-field defocus with +2.0 ± 11.1 *μ*m and the multifocal center-distance designed lens with +2.1 ± 11.1 *μ*m. This was followed by the MFN lens and the regular SVDC lens, where the choroid increased by +1.6 ± 11.3 *μ*m and +0.9 ± 11.2 *μ*m, respectively.

**Fig 5:**
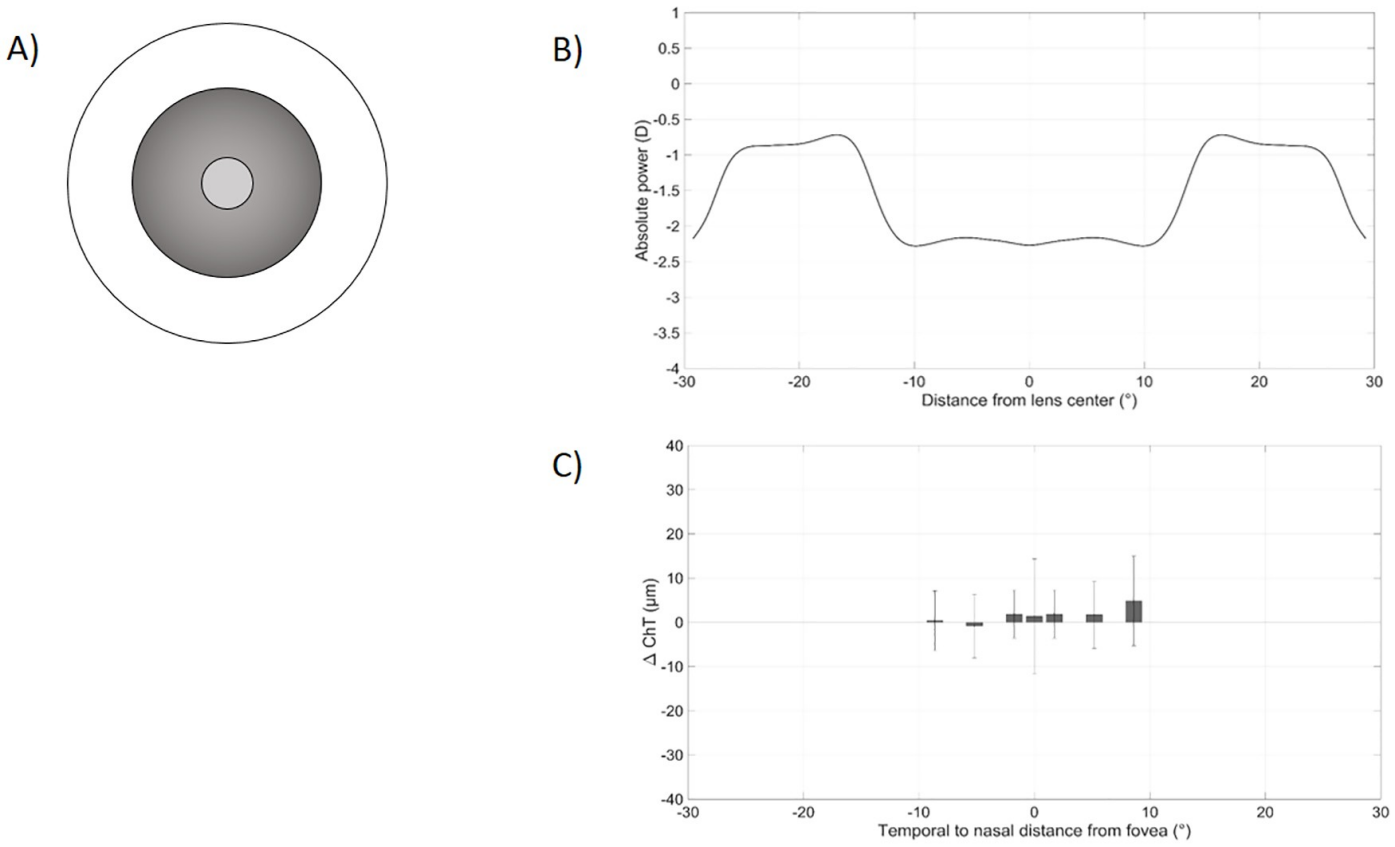
MFD lens profile compared with choroidal thickness changes. (A) Concentric structure of the MFD lens consisting of a 2.3 mm center with distance prescription and a progressive addition zone up to 8.5 mm. (B) Horizontal power profile of the MFD lens as measured and adapted from Wagner et al. [25]. (C) Changes in choroidal thickness from temporal to nasal retina after wearing the MFD lens for 30 min in the present study. The bars represent the temporal perifoveal, temporal parafoveal, central, subfoveal, central, nasal parafoveal and nasal perifoval retinal ETDRS area from left to right.

**Fig 6:**
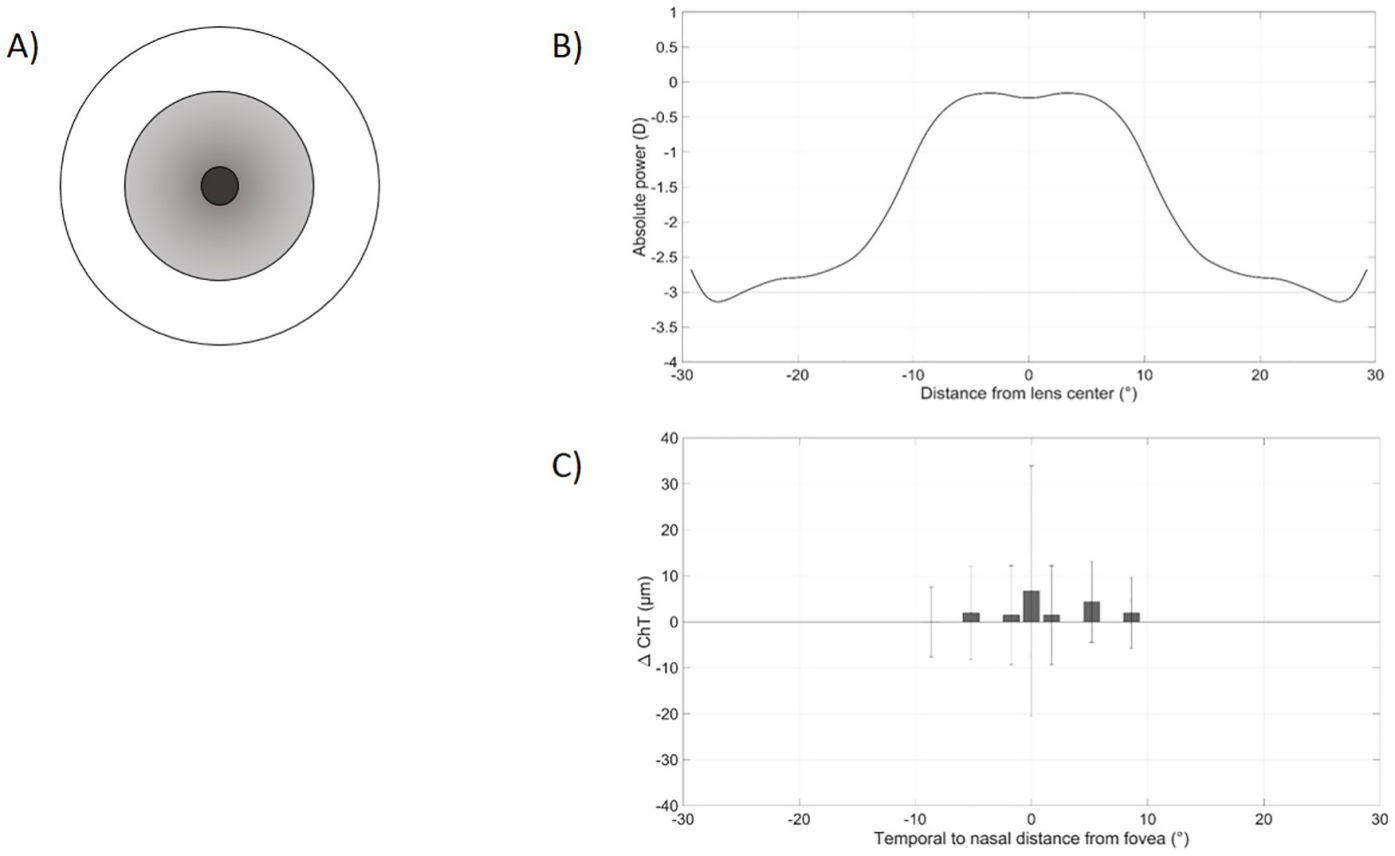
MFN lens profile compared with choroidal thickness changes. (A) Concentric structure of the MFD lens consisting of a 1.7 mm center with distance prescription and a progressive addition zone up to 8.5 mm. (B) Horizontal power profile of the MFN lens as measured and adapted from Wagner et al. [25]. (C) Changes in choroidal thickness from temporal to nasal retina after wearing the MFN lens for 30 min in the present study. The bars represent the temporal perifoveal, temporal parafoveal, central, subfoveal, central, nasal parafoveal and nasal perifoval retinal ETDRS area from left to right.

To visualize the optical design of an MFD or MFN lens and its influence on choroidal thickness, Figs 5 and 6 contain theoretical designs of the contact lenses as measured by Wagner et al. [25] and the subsequently measured changes of the ChT observed during the course of the present study. The power of the applied multifocal contact lenses changes from the center to its periphery and therefore imposes different levels of retinal defocus at different retinal locations. Regarding the MFD lens in Fig 5C, there is a tendency towards a higher increase of +4.9 ± 7.5 *μ*m in choroidal thickness towards the progressive zones on the nasal parafoveal side only. Conversely, the MFN lens elicits changes in choroidal thickness that show more similarities to the power profile of the contact lens, as displayed in Fig 6C. The myopically defocused subfoveal choroid seems to thicken more than the parafoveal and perifoveal regions with +6.7 ± 27.2 *μ*m vs. amplitudes ranging from 0.0 ± 7.6 *μ*m to 4.4 ± 8.8 *μ*m in the temporal perifoveal, respectively, in the nasal parafoveal retina.

**Fig 7:**
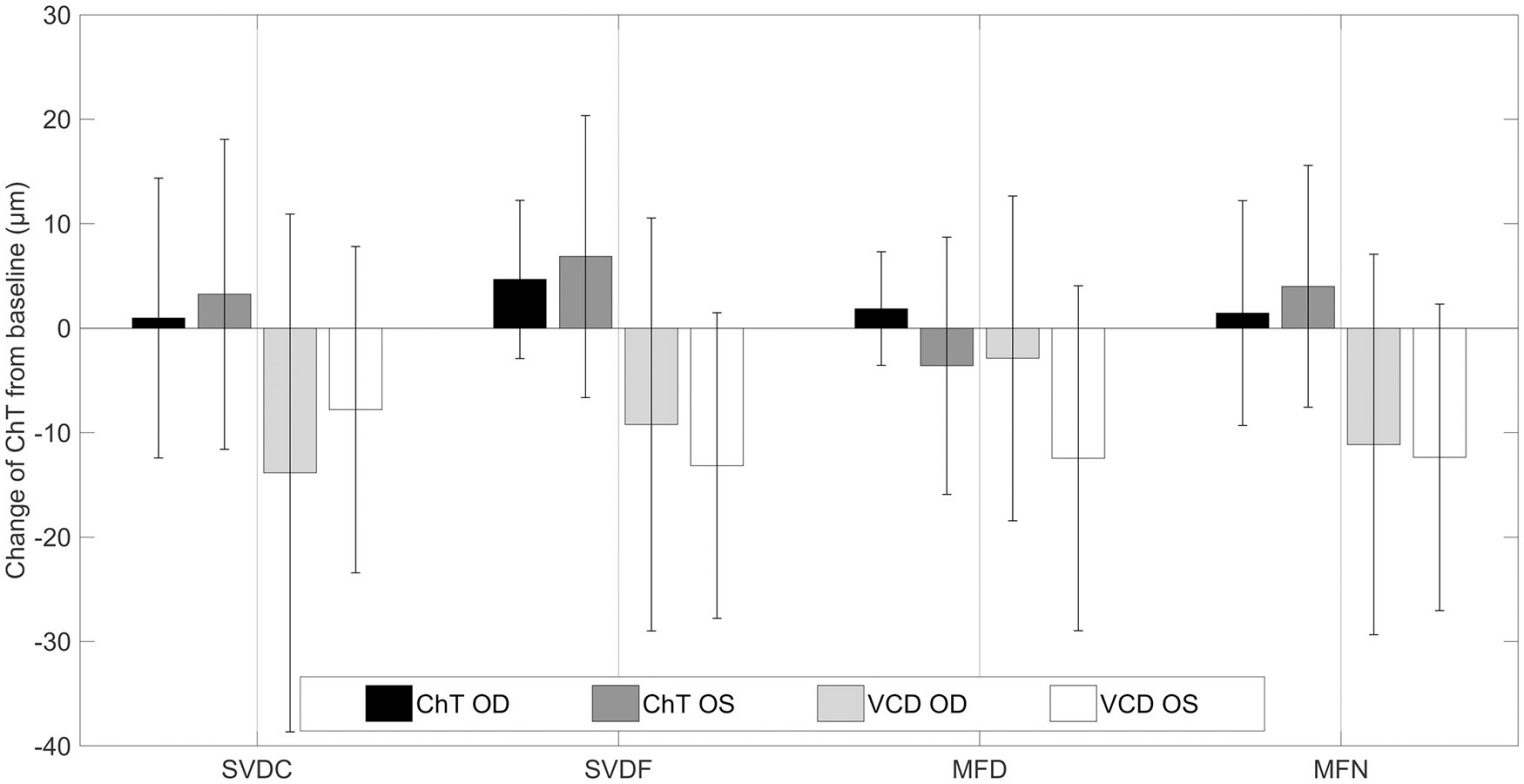
Choroidal and vitreous chamber depth (VCD) changes. Comparison of the lens-induced changes on the central ChT and vitreous chamber depth (VCD) between both eyes for every applied contact lens. General shortening from baseline of the vitreous chamber depth versus thickening of the choroid, whereas the left eye most commonly showed higher response rates. Errorbars indicate the standard deviation.

Using the left eye as a control eye, every point-location in each ETDRS area was compared with the corresponding point-location of the right eye. Statistical analysis revealed significant differences in ChT changes between both eyes for every contact lens and retinal point location. However, when analyzed separately, these significant changes had no consistent behavior across all subjects. This, in turn, did not allow reliable conclusions about the presence or absence of an intercular transfer. Neither was possible regarding the influence on vitreous chamber depth. Although vitreous chamber depth shortened in both eyes with all types of contact lenses, there were no statistically significant differences between the lenses or between both eyes (all p > 0.05). However, the left eye showed overall higher amplitudes of change than the right eye, except for with the SVDC lens. In the right eye, the central vitreous chamber depth shortened most with the SVDC lens by -13.9 ± 24.8 *μ*m and least with the multifocal center-distance designed lens by -2.9 ± 15.6 *μ*m. However, the central choroidal and vitreal reactions occurred approximately in the anti-phase to each other, as shown in Fig 7. Despite the overall opposite effects of vitreous chamber depth shortening with a central choroidal thickening, there was no significant correlation factor between the changes in ChT and vitreous chamber depth.

## Discussion

The purpose of this study was to investigate whether the protective effect of multifocal contact lenses can be attributed to an increase in choroidal thickness due to the imposition of myopic defocus on the retina via the addition zones within the lenses.

### Changes in choroidal thickness in response to contact lens wear

The main study illustrated, that short periods of wearing different single vision and multifocal contact lens designs lead to only small and nonsignificant changes in the thickness of the choroid and the vitreous chamber depth. However, it was observed that the changes in choroidal thickness versus vitreous chamber depth occurred in anti-phase to each other. No constant patterns were found that imply or rule out the presence of an interocular transfer.

When compared to other studies that impose full-field defocus via contact lenses, the resulting choroidal thickness changes in this experiment are less pronounced with all types of lenses [15, 16]. Although these studies solely investigated the effect of contact lens-induced full-field defocus between +2.0 D and +3.0 D, which matches best to our SVDF lens, they found choroidal thickness changes that are at least twice as high—with an exposure time twice as long as in the present study. The same accounts for comparisons with experiments that impose full-field defocuses ranging from +3.0 D to +5.0 D with spectacle lenses [12–14, 26]. Wang et al. (2016) [14] were the only group that did not observe absolute choroidal thickening with myopic defocus but instead relatively less thinning compared to the control eye. These discrepancies with past results might be caused by different study protocols and analysis methods in the present study. First, each session of contact lens wear was set to only 30 min instead of the commonly used 60 min, since significant changes of choroidal thickness with myopic defocus were already reported after 20 min of exposure with a +2.0 D defocusing contact lens [15], which we could not observe. Regarding the analysis methods, our study group was also the only group to evaluate choroidal changes across a larger retinal area containing the whole macula. With this, a broader overview of the thickness changes was possible, in contrast to the sole assessment of the subfoveal thickness or certain anatomical points along a horizontal cross-section through the fovea. This was achieved using fully automated choroidal segmentation software [21]. An additional advantage of this software, compared to manual segmentations, represents the analysis without influence of the examiner.

The original research question about the effect of the optical design of multifocal contact lenses on choroidal thickness can be answered to a certain extent in the present study. In fact, multifocal contact lenses were not able to induce significant thickness changes after short-term wear. However, it is also possible that the choroid does not react by a spontaneous thickness increase to myopic defocus only. As indicated in the complementary studies, the effect on choroidal thickness with multifocal contact lenses became more reinforced with long-term wear. These preliminary results suggest that the lenses not only prevent choroidal thinning during the day but are also able to influence its diurnal rhythm of thickness fluctuations. This is supported by the small amplitudes of thickness change (in the range of microns) that are insufficient to truly compensate the shifted image plane from a refractive point of view. Such defocus-induced phase shifts of the diurnal thickness rhythm have been previously described in animals and humans and have the potential to positively regulate axial growth [27, 28]. As recently shown, the choroidal thickness can also be influenced by the difference of ON to OFF signals of the viewed scene. More ON stimulation, such as reading black text on white background, leads to choroidal thinning, whereas more OFF stimulation, which occurs during reading white text on a black background, resulted in choroidal thickening [29]. In the present study, the average difference of the summed ON and OFF signals of the watched movie in the present study resembled natural viewing scenes and therefore did not provoke additional choroidal changes.

### Limitations

The findings might have also been influenced to a certain extent by the limitations of the experiment. First of all, the main study evaluated only short-term effects of multifocal contact lens wear, which does not truly reflect the long-term wear of such lenses over months or even years. The small complementary studies already implied slightly different results after eight hours with the lenses compared to sessions of 30 minutes. Moreover, this work only focused on distance viewing and did not include near activities with the multifocal lenses, which might reveal a greater impact on choroidal thickness. Regarding the acquisition and analysis procedures, the high repeatability index of the choroidal thickness measurement plus its segmentation of 10 *μ*m exceeds the observed effect sizes of approximately 2 *μ*m. Another important factor to consider is the centration of the multifocal contact lens on the eye. Although the lens centration was controlled in every session, the multifocal contact lenses could possibly move excessively or decenter for a certain amount of time without being noticed. These fluctuations are therefore able to affect both the location and amplitude of the choroidal responses, as well as the vitreous changes. Another important fact to consider is the area of the actually measured retina versus the area that is covered by the zones of the contact lens. The scanned choroidal area corresponds to the center-distance zone of the MFD lens due to its structurally wider center-distance zone up to ±12°. Despite the slight myopic defocus of -0.5 D in the corrected central part of the contact lens, as seen in Fig 5, there is almost no influence of additional myopic defocus from the progression zone of the lens. Conversely, the smaller center-near zone of less than 10° of the MFN lens and the subsequent progressive zone allowed analyses of choroidal thickness in areas that received visual input from the progressive zone of the MFN lens as well.

## Conclusion

In conclusion, the study did not show significant changes in choroidal thickness with different designs of multifocal contact lenses. However, a complete exclusion of choroidal involvement is not possible due to the visible trends of choroidal thickening and changed diurnal rhythm with the lenses. The long-term choroidal thickness changes during its diurnal rhythm are of particular interest lately [30] and should be investigated under the influence of different myopia control interventions, such as multifocal contact lenses. Since this work was restricted to distance viewing only, upcoming studies should determine whether there are more obvious changes in choroidal thickness with multifocal contact lenses during near viewing.

Moreover, the present study performed the first step in the direction of using fully automated choroidal segmentation software with the possibility of analyzing a broader area of choroid in the macula. As wide-field OCT scans can currently be performed, the evaluation of the peripheral choroid with associated thickness changes can be a next step in upcoming studies [30]. If used, off-axis optical parallaxes have to be considered due to the distance between the contact lens on the cornea and the size of the entrance pupil affecting the resulting peripheral refractive power of the contact lens, as well as the choroidal responses. It would also be oversimplifying to consider the visual output from multifocal contact lenses as solely myopic defocus, similar to a low-pass filter. Rather, these types of lenses result in a more complicated retinal aberration profile.
